# *Trichinella* infection in Serbia from 2011 to 2020: a success story in the field of One Health

**DOI:** 10.1017/S0950268823000109

**Published:** 2023-01-19

**Authors:** Sasa Vasilev, Ivana Mitic, Milorad Mirilovic, Dragana Plavsa, Emina Milakara, Budimir Plavsic, Ljiljana Sofronic-Milosavljevic

**Affiliations:** 1 National Reference Laboratory for Trichinellosis, Institute for the Application of Nuclear Energy – INEP, University of Belgrade, Belgrade, Serbia; 2 Faculty of Veterinary Medicine, University of Belgrade, Belgrade, Serbia; 3 Institute of Public Health of Serbia – Dr Milan Jovanovic Batut, Belgrade, Serbia; 4 Ministry of Agriculture, Forestry and Water Management of Serbia, Veterinary Directorate, Belgrade, Serbia; 5 World Organization for Animal Health (OIE), Paris, France

**Keywords:** Serbia, *Trichinella* infection, trichinellosis

## Abstract

In Serbia, modern pork production systems with implemented control measures, including the detection of *Trichinella* larvae in meat (ISO18743), have eliminated farmed pork from pigs slaughtered at abattoirs as a source of trichinellosis. Epidemiological data from 2011 to 2020 indicate that the number of human cases and the number of infected domestic pigs has decreased significantly. Over the years, pork was the most frequent source of human infection. Cases generally occurred in small family outbreaks, and the infection was linked to consumption of raw or undercooked pork from backyard pigs. In most of the outbreaks, *T. spiralis* was the aetiological agent of infection, but in 2016, a large outbreak was caused by consumption of uninspected wild boar meat containing *T. britovi* larvae. To achieve safe pork, it is important that consumers of pork from animals raised in backyard smallholdings and of wild game meat are properly educated about the risks associated with consumption of untested meat. Laboratories conducting *Trichinella* testing should have a functional quality assurance system to ensure competency of analysts and that accurate and repeatable results are achieved. Regular participation in proficiency testing is needed.

## Introduction

Parasitic nematodes from the genus *Trichinella* are causative agents for the zoonotic disease trichinellosis, which is a serious human health risk. This genus comprises 10 species: *T. spiralis*, *T. nativa*, *T. britovi*, *T. murrelli*, *T. nelsoni*, *T. patagoniensis* and *T. chanchalensis* (encapsulating species), *T. pseudospiralis*, *T. papuae* and *T. zimbabwensis* (non-encapsulated species) as well as three genotypes (*Trichinella* T6, T8 and T9) [1]. These nematodes are distributed worldwide and infect a broad variety of species while transmitting in both domestic and sylvatic cycles [2]. Consequently, *Trichinella* spp. infections in animals induce a food safety problem since humans acquire trichinellosis by the consumption of raw or undercooked meat of infected animals. In the EU, *Trichinella* spp. infection is well controlled since all *Trichinella*-susceptible animals intended for human consumption are required to be tested for the presence of *Trichinella* larvae in the muscles (Commission Implementing Regulation (EU) 2015/1375). Currently, trichinellosis is successfully kept under control in Serbia, as only individual cases occur annually [3]. Communication between medical practitioners, public health and veterinary authorities (One Health concept) contributed to this success. In Serbia, meat inspection to detect *Trichinella* larvae is mandatory, by using the digestion method (for meat from farmed domestic pigs in abattoirs) or either the digestion method or trichinelloscopy (for meat intended for private consumption and from backyard pigs on smallholdings). For testing meat from wild boars, only the digestion method is allowed. The aims of this study were to determine the prevalence of *Trichinella* infection in domestic pigs and wild boars in Serbia during 2011–2020 and to evaluate the status of human disease caused by *Trichinella* spp. in the same period.

## Materials and methods

### Data sources

Epidemiological and parasitological data regarding animal and human *Trichinella* infections in Serbia for the period 2011–2020 were collected and analysed.

Annual reports issued by the Veterinary Directorate of the Republic of Serbia, containing data on the number of slaughtered, inspected and infected pigs, as well as the number of hunted and infected wild boars, were used as the source of information for estimating the prevalence of *Trichinella* infection in animals. The Veterinary Directorate also provided data on backyard pigs from smallholdings for 2020.

Data pertaining to the annual rate of human trichinellosis, i.e. the number of outbreaks (including number of illnesses for each outbreak) and individual cases, as well as the sources of infection, were provided by the Institute of Public Health of Serbia Milan Jovanovic Batut, Serbia.

### Statistical analyses

We conducted a descriptive statistical analysis of epidemiological variables collected for 2011–2020 including traceback investigation information. We conducted a trend analysis using a linear trend and regression analysis. For comparing frequencies, the Chi-square (*χ*^2^) test was used. The differences in results were considered as statistically significant at the level of *P* < 0.05. Statistical analysis of the obtained results was carried out using statistical software GraphPad Prism version 6.00 for Windows (GraphPad, San Diego, CA, USA) and Microsoft Office Excel 2010 (Microsoft Corp., Redmond, WA, USA).

## Results

### *Trichinella* infection in domestic pigs

The average number of pigs slaughtered for human consumption, i.e. inspected pigs, in Serbia was, on average, 2 172 165 per year in the last 10-year period (minimum in 2012 – 1 507 182, and maximum in 2019 – 2 529 564). Data regarding the number of inspected and infected pigs for 2011–2020 are summarised in Table 1. During these years, an 11-fold reduction in the prevalence of *Trichinella* infection in pigs was observed. In 2011, *Trichinella* larvae in meat (muscle) were detected in 523 carcasses of inspected pigs, and the number constantly decreased during the next few years, reaching the lowest number of 47 infected carcasses in 2020 (statistically significant difference, *P* < 0.0001). The rate of infection in farmed domestic pigs in Serbia in 2020 was 0.002%, indicating successful maintenance of a low infection prevalence, which was quite reduced compared to the rates in 2011–2013 (the prevalence of 0.026% in 2011 trended downwards in the next 2-year period, reaching the prevalence of 0.014% in 2013). The prevalences calculated for 2014–2017 corresponded to the prevalences that were measured in Serbia in the years before 1980 (<0.009%).

**Table 1.** *Trichinella* infection in pigs in Serbia, 2011–2020

	Year									
	2011	2012	2013	2014	2015	2016	2017	2018	2019	2020
No. of inspected pigs (×1000)	1970	1507	2037	2142	2221	2319	2314	2432	2529	2248
No. of infected pigs	523	299	292	148	133	178	111	71	64	47
Per cent of inspected pigs that were infected	**0.026**	**0.020**	**0.014**	**0.007**	**0.006**	**0.008**	**0.005**	**0.003**	**0.003**	**0.002**

**Fig. 1.**
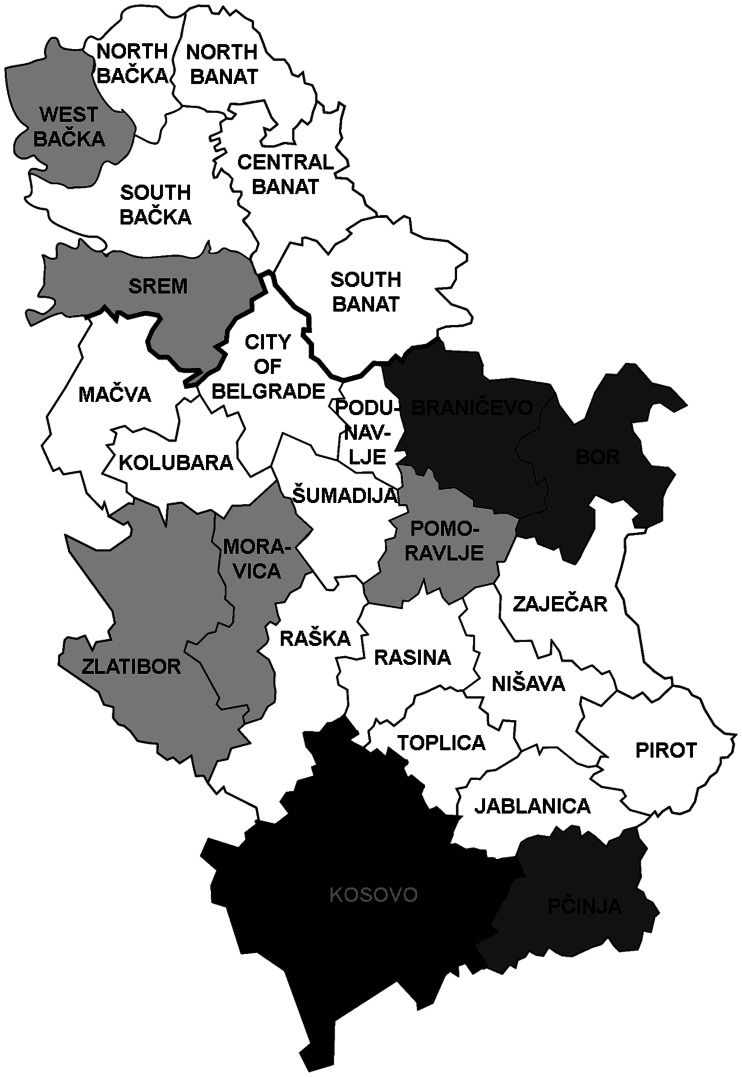
Geographical distribution and average prevalence of *Trichinella* infection in domestic pigs in 25 districts in Serbia, 2011–2020. Dark grey – districts with an average prevalence above 0.05%. Light grey – districts with an average prevalence of 0.01–0.03%. White – districts with an average prevalence under 0.01%. Black – no data available. Image source: https://en.wikipedia.org/wiki/Districts_of_Serbia#/media/File:Districts_of_Serbia.png.

During 2011–2020, *Trichinella* infection in pigs was detected in 23 out of 25 districts in Serbia (excluding Kosovo, because no data were available). Prevalences on average above 0.05% were found in Branicevo, Bor and Pcinja districts (Fig. 1), while for the other districts, prevalences on average during 2011–2020 were from 0.00% to 0.022%.

Improvements in pig rearing systems and the implementation of control measures [4–7] have eliminated *Trichinella* infection in pigs from controlled housing, and these farms now (2020) have no positive animals. In 2020, all *Trichinella-*positive pigs in Serbia were from backyard smallholdings with uncontrolled housing conditions (Table 2).

**Table 2.** Number of *Trichinella-*infected pigs in Serbia in 2020, classified according to housing conditions

All pigs	Pigs from non-controlled housing conditions	Pigs from controlled housing conditions
No. tested/No. positive/prevalence	No. tested/No. positive/prevalence	No. tested/No. positive/prevalence
2 248 239/47/0.002	112 346/47/**0.042**	2 135 893/0/0

### *Trichinella* infection in humans

**Fig. 2.**
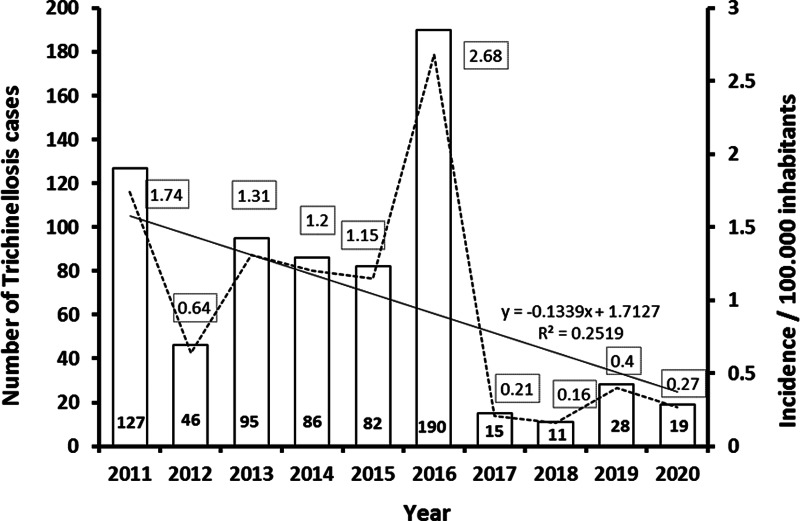
Number of trichinellosis cases per year in Serbia, 2011–2020, and trichinellosis incidence rate per 100 000 inhabitants, Serbia, 2011–2020. The trendline shows a steady decline in the incidence of human trichinellosis cases during this period. Trend line was derived by using the equation *y* = −0.1339*x* + 1.7127, based on data points in a Microsoft Excel chart, *R*^2^ = 0.12519.

**Fig. 3.**
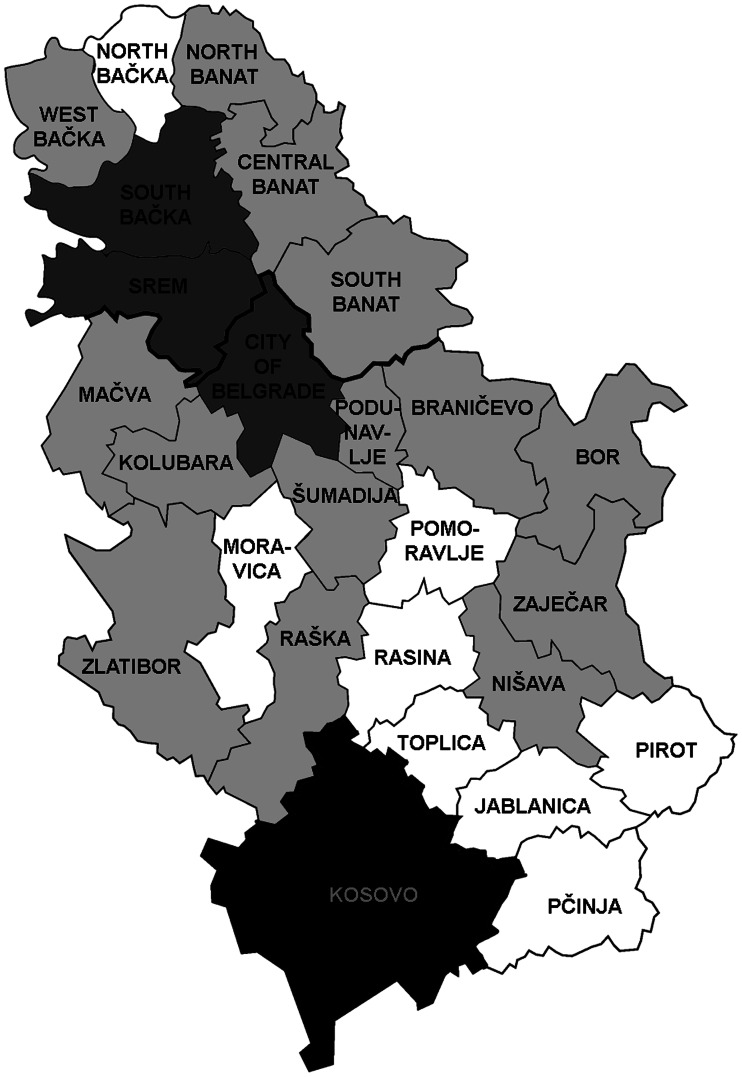
Human *Trichinella* spp. infection in 25 districts of Serbia, 2011–2020. Dark grey – districts with trichinellosis cases registered in 4–6 years of the 10-year study period. Light grey – districts with trichinellosis cases registered in 1–3 years of the 10-year study period. White – districts with no trichinellosis cases registered in the study period. Black – no data available. Image source: https://en.wikipedia.org/wiki/Districts_of_Serbia#/media/File:Districts_of_Serbia.png.

During 2011–2020, a total of 699 cases of trichinellosis, on average 69.9 per year, without deaths, were reported in Serbia (Fig. 2). The calculated incidence of human infection at the country level for this period is shown in Figure 2. The annual incidence of trichinellosis varied from 2.68 to 0.16 per 100 000 inhabitants during those 10 years. In three districts in Serbia, outbreaks were reported in 5–6 years, in 13 districts, outbreaks occurred rarely and only in 1–3 years during the period, while there were no cases of infection in eight districts from 2011 to 2020 (Fig. 3).

**Fig. 4.**
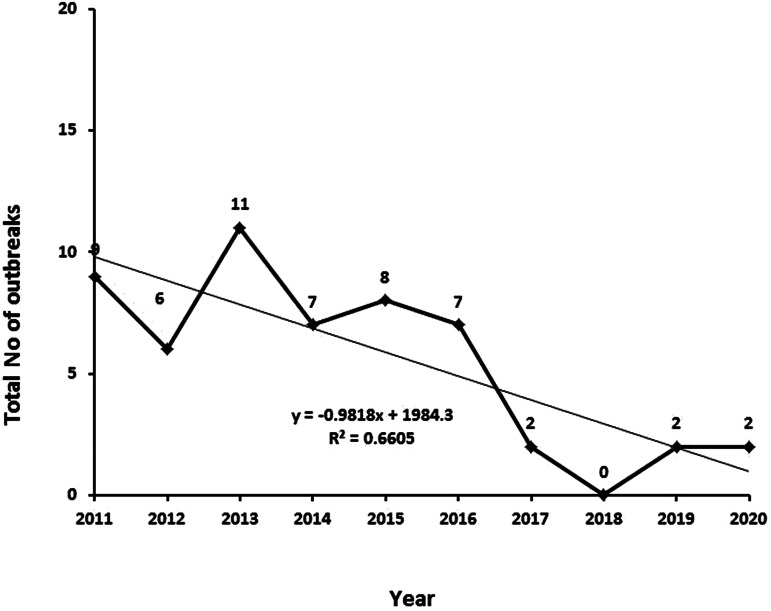
Number of trichinellosis outbreaks per year in Serbia, 2011–2020. Trend line was derived by using the equation *y* = −0.9818*x* + 1984.3, based on data points in a Microsoft Excel chart, *R*^2^ = 0.6605.

A total of 54 outbreaks occurred in Serbia during 2011–2020 (Fig. 4). They were distributed equally between urban and rural areas. Epidemiological investigations performed in this period revealed that different sources of infection were responsible for the outbreaks of trichinellosis. The cause of the infection in 37 (88.09%) of the outbreaks was the consumption of pork, specifically raw or undercooked meat or meat products (homemade sausages and smoked meat). Other sources of infection included horse meat (1 outbreak, accounting for 2.38% of the outbreaks, and which took place in 2014) and wild boar meat (4 outbreaks, 9.52%, which took place in 2012, 2013, 2015 and 2016).

### *Trichinella* infection in wild boars

**Fig. 5.**
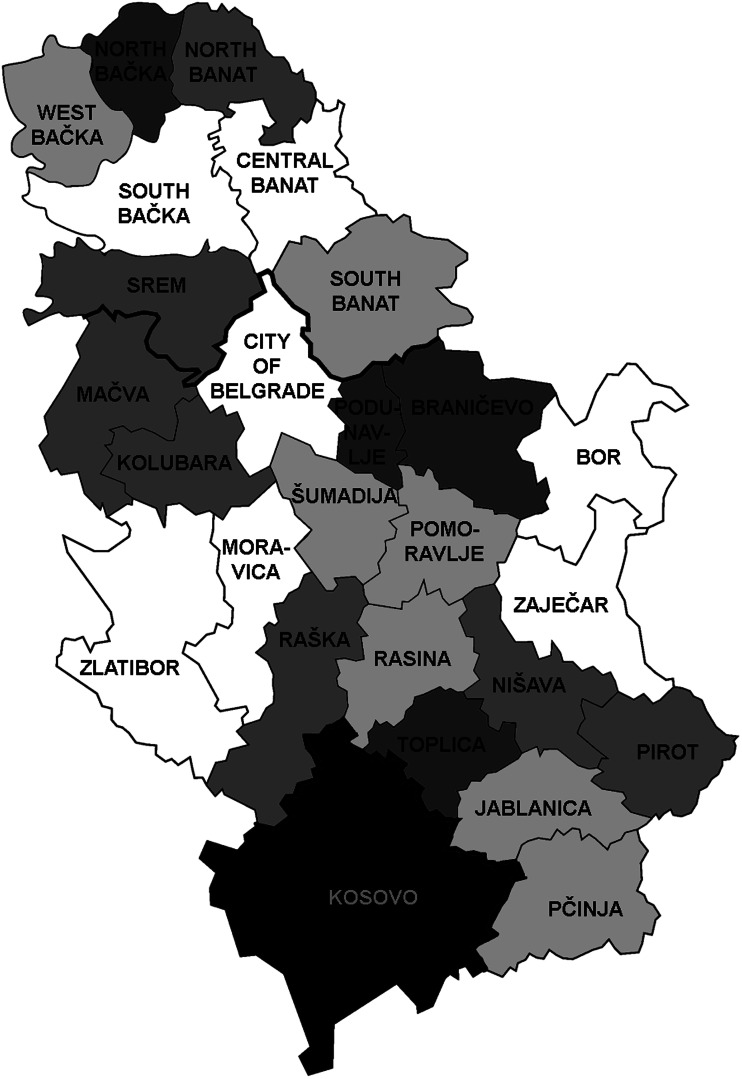
Geographical distribution and average prevalence of *Trichinella* infection in wild boars in 25 districts in Serbia, 2011–2020. Dark grey – districts with an average prevalence above 3%. Grey – districts with an average prevalence of 1–3%. Light grey – districts with an average prevalence of 0.5–1%. White – districts with an average prevalence of under 0.5%. Black – no data available. Image source: https://en.wikipedia.org/wiki/Districts_of_Serbia#/media/File:Districts_of_Serbia.png.

Over the past 9 years (compared with the situation previously), parasitological testing by digestion of wild boars in game-handling establishments became more intensified and provided better insight into the prevalence and distribution of *Trichinella* in these animals (Fig. 5). The number of districts in which wild boars were hunted, tested and reported for *Trichinella* increased during the time (from 10 districts in 2012 up to all 25 districts in 2015), showing, nowadays, the reliable prevalence of *Trichinella* infection of 0.800% and 1.087% (in 2019 and 2020, respectively) and a prevalence range in the last 10 years from 2.32% in 2012 to 1.087% in 2020 (Table 3). Speciation of *Trichinella* was performed sporadically, and only the existence of *T. britovi* and *T. spiralis* infection was recognised among wild boars.

**Table 3.** *Trichinella* infection in wild boars in Serbia for the period 2011–2020

	Year								
	2012	2013	2014	2015	2016	2017	2018	2019	2020
No of inspected wild boars	1892	3827	4814	7085	8650	9560	15 104	12 997	11 956
No of infected wild boars	44	77	61	52	94	90	84	104	130
Per cent of animals infected	**2.320**	**2.012**	**1.267**	**0.737**	**1.087**	**0.948**	**0.556**	**0.800**	**1.087**

## Discussion

*Trichinella* infection in animals and humans in Serbia is still recognised as a health and animal husbandry problem, and some regions endemic for this zoonosis persist in the country, although a significant effort has been made to reduce the infection spreading and even to eliminate the parasite from the food chain. A promising trend of decreasing prevalence of *Trichinella* infection in pigs in Serbia was achieved during 2011–2020. The observed decrease in the infection rate in pigs could be attributed to implementation of animal health measures by veterinary services introduced from 2006 [8].

The prevalence of *Trichinella* infection in pigs is better than the prevalences seen during the 1980s, before the re-emergence of trichinellosis in this country [8–10], and is better than the prevalences measured in 2001–2010 [8]. Although the prevalence of infection in domestic pigs has a downward trend, the efforts spent on reducing the spread of infection did not reduce the area where it is present, since *Trichinella* was detected in pigs over almost all of Serbia during 2011–2020. This observation implies that some further improvements to hygienic and sanitary conditions on farms and by households with backyard pigs should be implemented; this refers primarily to rat control programmes, since rats are considered as important vectors in the transmission of *Trichinella* infection to domestic pigs [5, 6, 8].

Along with the significant decline of the *Trichinella* prevalence in pigs, the number of human infection cases detected during last 10 years was also significantly lower than during 2001–2010. The distribution of human infection across the country corresponded to the geographical distribution of *Trichinella* infection in domestic backyard pigs that are now the main source of infection. This indicates that in districts where trichinellosis outbreaks occurred more frequently, there is inadequate awareness of the risk from consumption of untested wild boar meat and/or of meat from smallholdings with backyard and/or free-ranging pigs. In 2011–2020, the incidence of human cases was lower than during the previous decade, and altogether, the infection rates for the current 10-year period correlated with those detected during the 1980s. Since most of the outbreaks in Serbia were pork-related, it is worth mentioning that some of the observed causes of human infection, discussed previously by Sofronic-Milosavljevic *et al*. [8], still exist: the lack of knowledge that causes the reluctance of some individual farmers to provide meat samples for *Trichinella* testing to the veterinary service; and insufficient training and experience of a part of the veterinary inspection staff. New measures introduced by the Veterinary Directorate are additional education of veterinarians and certification for *Trichinella* testing. Also, from 2022, it is mandatory for all analysts testing meat for *Trichinella* to participate in proficiency testing (PT). For the first time in Serbia, PT for the detection of *Trichinella* larvae in meat by the magnetic stirrer method was organised in 2017 by NRLT INEP and according to the rules for PT providers. All participants (veterinary institutes) successfully passed the PT [11]. In the years that followed, veterinary institutes achieved good results in PT organised in 2021 by NRLT INEP [12], and in 2022 by the Faculty of Veterinary Medicine, University of Belgrade (personal communication). The first *Trichinella* PT (NRLT INEP was the provider) for laboratories in export abattoirs was administered in 2022, and all participant laboratories successfully met the criteria (NRLT INEP data).

Molecular analysis for species identification has been sporadically performed on samples from infected Serbian pigs (at NRLT-INEP, and EURLP, ISS, Rome, Italy), revealing that only *T. spiralis* is present in domestic pigs in Serbia [8, NRLT-INEP data] out of the four *Trichinella* species (*T. spiralis*, *T. britovi*, *T. pseudospiralis* and *T. nativa*) identified in Europe so far [13]. In the last 10 years, we have seen a significant improvement in the human incidence of trichinellosis, and the number of infected people is at the historically lowest level. In Serbia, the consumption of pork and products thereof originating from farms with controlled housing conditions, good rearing systems and all control measures implemented, including routine mandatory *Trichinella* testing by artificial digestion at public (belonging to veterinary institutes) and private (at veterinary stations and abattoirs) laboratories, does not present a risk for human infection. However, consumption of uninspected and undercooked meat originating from backyards and from locally slaughtered domestic pigs on private properties is considered an important risk for human infection with *T. spiralis*. Usually, the people involved in the processing of meat are affected, as are their families and people to whom infected meat is gifted, so most of the outbreaks are small family epidemics [8]. In addition, in Serbia, wild boars also present a serious risk of *Trichinella* spp. transmission to humans.

Despite the implementation of mandatory measures for controlling *Trichinella* in pork, small outbreaks of trichinellosis and some sporadic cases still occur in Serbia [4]. At the beginning of 2017, there were cases of imported (in personal luggage) untested, infected meat delicacies (as a gift) from Serbia to France and shared with about 47 relatives and friends, so a total of 20 cases of trichinellosis were reported − nine in France and 11 in Serbia [14].

According to the number of human infection cases that have persisted over the last decade, it can be concluded that the above mentioned risk factors are still present in Serbia and that they continue to influence the occurrence of human cases. It is encouraging that the number of districts where trichinellosis occurred for 4–6 years during 2011–2020 decreased from 8 to 3 out of 25, and the number of districts with no human cases during the same period increased from 6 to 8. This distribution mostly corresponded to the geographical distribution of *Trichinella* infection in domestic pigs. This indicates that in districts where trichinellosis outbreaks occur more frequently, there is inadequate awareness of the risk of infection that can exist on smallholdings with backyard and/or free-ranging pigs. An exception was observed for Pcinja district, where there was a high prevalence of pig infection, but with no human cases detected during that period, which could be interpreted as being due to the effectiveness of control measures and public education conducted in this part of the country.

The One Health success in the field of *Trichinella* spp. control becomes evident when comparing the data presented here with those published for 2001–2010. In that decade, 2257 human infection cases, including three deaths, occurred. During that 10-year period, there were 144 outbreaks – 87 in the first 5 years and 57 in the second 5-year period [8]. In 2011–2020 (the current study), the total of 699 human cases in Serbia was three times lower than in the previous decade. Moreover, a total of 54 outbreaks were registered. In 2021, no cases of trichinellosis were registered, and in 2022, one outbreak with 26 confirmed cases of trichinellosis has been registered so far.

Meat and meat products offered to relatives and friends can be a source of infection with *Trichinella* when backyard pigs are raised without any compliance with hygienic rules and carcasses are not tested for *Trichinella* infection after slaughter [15]. Over the years, pork was the most frequent source of trichinellosis in Serbia. Cases generally occurred within family outbreaks, and the risk derived from consumption of untested backyard pork [3]. In most trichinellosis outbreaks in Serbia, *T. spiralis* was the aetiological agent of infection, but in 2016, a large outbreak was caused by consumption of wild boar meat containing *T. britovi* larvae [16, 17]. This was in spite of *T. spiralis*-infected pork meat being considered as the main source of the disease in humans. Alongside the reduction of the infection prevalence in pigs, it has become obvious that some cases of trichinellosis originate from the consumption of infected game meat. In 93% of outbreaks reported in 2011–2020, the source of infection was undercooked pork meat or meat products, while infected wild boar meat and horse meat were identified as the sources in 5% and 2% of outbreaks, respectively.

Wild boars are a significant reservoir of *Trichinella* spp. infection with consequent risk for human infection, and three outbreaks during the last decade were caused by the consumption of infected wild boar meat. Importantly, though, during the last decade, the inspection of wild boar meat was intensified, and now, wild boar meat from all 25 of the districts in Serbia is subjected to *Trichinella* inspection. The latest data have shown that all of the districts in Serbia host wild boars that have *Trichinella* infection. This implies that awareness of the necessity of meat inspection must be increased among hunters and potential consumers of wild boar meat.

The One Health approach to achieving *Trichinella* spp. infection control in Serbia is realised through the cooperation of all stakeholders at the local, regional and European levels. Medical practitioners who notice a case of trichinellosis immediately alert the Public Health Institute and the Veterinary authority, because effective communication, reporting and trace back system are necessary to identify the source of infection and prevent the spread of infection. The Veterinary Directorate in Serbia has introduced *Trichinella* control measures, such as systematic pig identification and registration systems, mandatory testing for *Trichinella* infection in all pigs slaughtered for human consumption (regardless of whether this is in abattoirs or on smallholdings), surveillance of wild boar populations and mandatory rodent control on pig farms all over the country. However, intensified supervision of the control measures was implemented in the areas with the highest infection prevalence [4, 8]. All these measures had a One Health effect, reducing the number of infected animals, and consequently, reducing the number of trichinellosis cases, which was noticeable from year to year. The public is now informed about trichinellosis in local newspapers, radio stations and televisions, as well as on posters displayed in local veterinary stations, Public Health Institutes and health centres; these posters show proper sampling and transport of meat, along with the reasons why it is necessary to inspect meat for the presence of *Trichinella* spp. In addition to the activities described above at the Serbian (national) level, there is cooperation at the European level, and NRLT INEP is recognised in the network of parasitological laboratories of the Istituto Superiore di Sanità (ISS, Rome, Italy) (https://www.iss.it/en/web/guest/eurlp-chi-siamo).

As a result of improvements in pig production on farms and application of mandatory *Trichinella* inspection, the percentage of *Trichinella*-infected pigs decreased over the period 2001–2010 [8]. The trend of a reducing *Trichinella* prevalence in pigs continued in 2011–2020 with a statistically significant reduction, finally reaching the value of 0.002% in 2020.

Until 2009, *T. spiralis* was considered as the only species from the genus *Trichinella* that was present in domestic and wild animals in Serbia. Molecular analyses enabled the detection of *T. britovi* in red foxes and wolves for the first time in 2009 [18], in addition to the already confirmed presence of *T. spiralis* in wildlife [13]. These analyses were the beginning of extensive study on *Trichinella* species in domestic pigs and wildlife. Notably, 6% of 469 animals hunted in Serbia between 1994 and 2013 were infected with *Trichinella*, showing that wild carnivores are important for *Trichinella* transmission in both the sylvatic and domestic cycles [19]. In Serbia, half (49.5%) of the examined wolves were infected with *T. britovi* [20], while 16.5% of golden jackals were infected with *Trichinella*, of which more than 2/3 had *T. spiralis* and 1/3 had *T. britovi* [21]. These investigations have shown there is a risk that several species of wild animals can transmit *Trichinella* spp. to the backyard and free-ranging pig populations and, thus, contribute to the maintenance and spread of the parasite.

## Conclusion

Although it is obvious that implementation of veterinary measures in Serbia has resulted in a considerable reduction of *Trichinella* infections in pigs, annually repeating outbreaks of trichinellosis indicate insufficient awareness of the risk of the disease and suggest that further efforts should be made in terms of education and trichinellosis prevention (using the One Health concept). To achieve safe food for consumers, it is important that: (1) consumers of meat from backyard pigs and hunters/consumers of wild game meat should be educated about the risk associated with consumption of untested meat; (2) laboratories conducting *Trichinella* testing should have a functional quality assurance system that ensures analyst competency and that accurate and repeatable results are achieved. Regular participation in PT is needed.

## Data Availability

This manuscript describes data obtained from the Veterinary Directorate and the Institute for Public Health of Serbia, and as such there is no broader dataset for release. Some data that support the findings of this study are publicly available from the Institute for Public Health of Serbia website. Any queries regarding the data can be directed to the corresponding author.
